# Effects of transcranial direct current stimulation and mindful walking on directional brain connectivity: An isolated effective coherence analysis

**DOI:** 10.1016/j.cnp.2026.07.006

**Published:** 2026-07-13

**Authors:** Keiichiro Nishida, Naoya Onishi, Yosuke Koshikawa, Tomonari Yamane, Toshiyuki Shimizu, Shunichiro Ikeda, Daisuke Haruna, Masafumi Yoshimura, Tetsufumi Kanazawa

**Affiliations:** a Department of Neuropsychiatry, Faculty of Medicine, Osaka Medical and Pharmaceutical University, Takatsuki, Osaka, Japan; b Department of Neuropsychiatry, Faculty of Medicine, Kansai Medical University, Hirakata, Osaka, Japan; c Department of Occupational Therapy, Faculty of Rehabilitation, Kansai Medical University, Hirakata, Osaka, Japan

**Keywords:** Default mode network, Depression, EEG LORETA, Effective coherence, Mindfulness, Resting state network, Transcranial direct current stimulation (tDCS)

## Abstract

**Introduction:**

Mindfulness and transcranial direct current stimulation (tDCS) modulate brain networks implicated in psychopathology. This study investigated the synergistic effects of concurrent anodal tDCS over the left dorsolateral prefrontal cortex (DLPFC) and treadmill walking for Focused Mindfulness (TW-FM) on effective directional brain connectivity for focused mindfulness.

**Methods:**

Seventy-seven healthy participants were assigned to three groups: Active tDCS with Mindful walking (Active-MW), Sham tDCS with MW (Sham-MW), or Active tDCS during Resting State (Active-RS) by combining data sets from two individual studies. Isolated effective coherence (iCoh) in the theta and alpha bands was analyzed using standardized low-resolution brain electromagnetic tomography (sLORETA).

**Results:**

Active tDCS significantly reduced information flow from the posterior cingulate cortex (PCC) to the rostral anterior cingulate cortex (rACC) within the Default Mode Network (DMN). Furthermore, the mindfulness intervention significantly attenuated the flow from the rACC to the medial prefrontal cortex (mPFC) and the flow from the left inferior parietal lobe to the mPFC. Notably, Active tDCS strengthened interhemispheric Executive Control Network (ECN) connectivity from the right to the left DLPFC. Conversely, the Active-MW group exhibited reduced alpha band connectivity from the right DLPFC to the rACC compared to the other groups.

**Conclusions:**

The combined intervention elicited hierarchical decoupling of the DMN. tDCS suppressed bottom-up emotional pathways, whereas mindfulness inhibited the propagation of salient signals to self-referential processing. Simultaneously, the intervention strengthened top-down executive control. These findings suggest that combining tDCS with mindful walking effectively ameliorates DMN-dominant/ECN-recessive imbalance., highlighting its potential as a clinical tool to facilitate mindfulness engagement.

## Highlights

• tDCS and mindfulness synergistically decoupled the default mode network.
• tDCS enhanced interhemispheric executive connectivity, promoting neural efficiency.
• This dual approach may correct network imbalance, normalizing pathological networks.

## 1 Introduction

Many studies have demonstrated that the brain activity during rest and task performance is organized into distinct functional networks (Fox et al., 2005; Yeo et al., 2011). The Triple Network Model proposed by Menon serves as a central framework for understanding the basis of human cognitive control and emotion regulation in this complex system. This model relies on interactions among three core networks: the Default Mode Network (DMN), which is involved in self-referential thinking and mind-wandering; the Executive Control Network (ECN), responsible for external tasks and cognitive control; and the Salience Network (SN), which mediates dynamic switching between these two antagonistic networks (Menon, 2011). Accumulating evidence suggests that in depression and anxiety, DMN hyperactivity is associated with increased rumination (Kaiser et al., 2015) (Hamilton et al., 2015), reduced top-down control by the ECN (Fox et al., 2006), and SN dysfunction. Thus, reconstructing the balance between these networks is a key therapeutic goal.

Mindfulness and transcranial direct current stimulation (tDCS) have garnered attention as approaches with synergistic effects, such as controlling emotion (Badran et al., 2017; Divarco et al., 2023; Wickens et al., 2025). Mindfulness is fundamentally defined as the awareness that emerges through paying attention on purpose, in the present moment, and non-judgmentally to the unfolding of experience (Kabat-Zinn, 2003). While foundational mindfulness practices typically begin by anchoring attention to somatic sensations (Kerr et al., 2013), the objective of mindful walking extends to cultivating a receptive, detached awareness not only toward these internal bodily sensations-such as the soles of the feet and body movements-but also toward thoughts and emotions arising in the moment, without labeling them (Davis et al., 2022). Essentially, it is a process of walking while paying attention to the senses, emotions, thoughts, and automatic behavioral patterns (Gotink et al., 2016). According to a review, mindfulness meditation enhances emotion regulation by strengthening prefrontal cognitive control mechanisms while simultaneously decreasing DMN activity (Tang et al., 2015a).

While anodal tDCS has traditionally been shown to enhance local cortical excitability (Nitsche and Paulus, 2000), recent neuroimaging studies demonstrate that stimulation targeting the left dorsolateral prefrontal cortex (DLPFC) actively modulates large-scale brain networks (Jamil et al., 2020; Meinzer et al., 2024), including the enhancement of ECN connectivity and attenuation of DMN hyperactivity (Keeser et al., 2011). Therefore, it is anticipated that concurrent mindfulness and tDCS may exhibit synergistic effects in reconfiguring these core networks. Empirically, our previous study demonstrated that combining treadmill walking for focused mindfulness (TW-FM) with tDCS reduced anxiety more effectively than either intervention alone (Nishida et al., 2021). However, the detailed neural mechanisms by which this synergistic effect modulates the information flow between brain networks remain unclear.

Conventional functional connectivity analyses are limited by their inability to identify the causal direction of the interactions between brain regions. Pascual-Marqui et al. pointed out the importance of eliminating the influence of indirect pathways in multivariate analyses (Pascual-Marqui et al., 2014). To address these issues, this study employed isolated effective coherence (iCoh), developed by Pascual-Marqui et al. and validated in attentional tasks (Kitaura et al., 2017) and in patients with mild cognitive impairment (Caravaglios et al., 2025), schizophrenia (Wada et al., 2020), and depression (Minami et al., 2022). iCoh quantifies the direct causal connectivity between regions in specific frequency bands, while excluding the effects of indirect pathways and instantaneous connections. This methodology allows for the elucidation of how tDCS and mindfulness generate “information flow” among the brain networks.

This study elucidated the effects of concurrent tDCS and mindful walking on the causal connectivity within large-scale brain networks. Specifically, leveraging two existing datasets from our group (Nishida et al., 2021; Nishida et al., 2019), this study was designed to delineate the distinct neurophysiological features associated with each intervention by comparing the three conditions: tDCS Active-Mindfulness Walking (MW) Group, tDCS Sham -MW Group, and tDCS Active-resting state (RS) Group.

## 2 Methods

### 2.1 Study design

To elucidate the distinct neurophysiological contributions of tDCS and TW-FM, the present study was conducted as a secondary analysis combining existing datasets from two independent previous studies, hereafter referred to as the CEED 1 study (Nishida et al., 2019) and the CEED 2 study (Nishida et al., 2021). Although the CEED 1 study included both patients with depression and healthy volunteers, and the CEED 2 study included healthy volunteers, the present analysis focused exclusively on healthy volunteers. In both original studies, these individuals underwent a single 20-min session of anodal tDCS (1 mA) targeting the left dorsolateral prefrontal cortex.

The CEED 1 study (Nishida et al., 2019) employed a within-subject crossover design to compare the effects of different brain stimulation sites during a resting state. In contrast, the CEED 2 study (Nishida et al., 2021) was a randomized, double-blind, sham-controlled trial investigating the combined effects of tDCS and TW-FM.

For the current secondary analysis, we extracted and reorganized the data from these two distinct trials to compare three conditions: the Active stim-MW group (active tDCS during TW-FM), the Sham stim-MW group (sham tDCS during TW-FM), and the Active stim-RS group (active tDCS during the resting state).

While the present analysis utilized the same participant datasets as reported in our previous publications (Koshikawa et al., 2022; Nishida et al., 2025; Nishida et al., 2021; Nishida et al., 2019), this investigation is entirely novel; we applied isolated effective coherence (iCoh) - a completely different analytical framework and outcome measure - to evaluate directional causal brain connectivity.

### 2.2 Participants

The participants were healthy right-handed adults **with no prior experience in mindfulness meditation.** Individuals with a history of psychiatric or neurological disorders, head surgery, or head injuries resulting in disorders of consciousness were excluded. Specific contraindications for electrical stimulation were also grounds for exclusion, including skin problems, a history of epileptic seizures, or syncope. Individuals who were pregnant or possibly pregnant, and those who had prior experience with neuromodulation, were excluded.

The study protocol described by Nishida et al. (2019) was approved by the Institutional Ethics Review Committee of Kansai Medical University and registered with the UMIN-CTR (https://www.umin.ac.jp/ctr/; registration Nr. UMIN000015046). The study by Nishida et al. (2021) was approved by the Certified Review Board of the Hyogo College of Medicine (approval number: CRB51800005) and registered with the jRCT (https://jrct.mhlw.go.jp/; registration Nr. jRCTs052180043). Both studies were conducted according to the Declaration of Helsinki. Participants were recruited through posters on the staff bulletin boards of Kansai Medical University and its affiliated institutes.

### 2.3 Procedures

The interventions were conducted in a single session. The timeline follows the procedures used in these studies.

1. Pre-measurement: Resting-state eyes-closed electroencephalography (EEG) recordings (5 min) were obtained and psychological scales were administered.
2. Intervention (20 min):

• Active stim-MW / Sham stim-MW groups: Participants performed TW-FM while walking on a treadmill. The use of a treadmill allowed participants to reliably maintain a constant slow walking speed (1 km/h), which is a key feature of this intervention. While walking, participants received the following audio instruction every two minutes: “Focus on the sensations in your soles. Heel to toe, heel to toe. Focus on shifting your weight from one thigh to the other. Then, any thoughts that arise will disappear as you walk.” This continuous prompting directed their attention toward present-moment somatic sensations, distinguishing this treadmill task as TW-FM (Nishida et al., 2021).
• Active stim-RS group: Participants did not perform the walking task; they received active tDCS while sitting in a chair in a silent EEG-protected room.

3. Post-measurement: Immediately after the intervention, resting-state eyes-closed EEG recordings and psychological scales were administered.

### 2.4 tDCS protocol

In both studies, a single session of tDCS was administered using DC-Stimulator Plus (NeuroConn, Ilmenau, Germany). The anode (5 cm in diameter, circular, 20 cm^2^) was placed over the left dorsolateral prefrontal cortex (DLPFC; F5), and the cathode (5 × 7 cm, rectangular) was placed on the left shoulder. For the active stimulation condition, a constant current of 1 mA was applied for 20 min, including 30-s ramp-up and ramp-down periods. For the sham stimulation condition, to maintain blinding, the current was ramped up to 1 mA over 30 s, maintained for 30 s, and then ramped down with no current delivered for the remaining time.

### 2.5 EEG recording and processing

EEG data were recorded for 5 min in a resting eyes-closed state before and after the intervention using an EEG-1200 system (Nihon Kohden, Tokyo, Japan). The sampling frequency was 500 Hz, recorded with a 0.5 Hz low-cut filter (time constant) and a 60 Hz high-cut filter.

• Active stim-RS group: Recorded using a 64-channel Ag/AgCl sintered Waveguard Original EEG cap (ANT Neuro, Netherlands). For the analysis, 30 channels were selected from the 64-channel recording after ensuring map smoothness through visual inspection by two skilled staff members.
• Active stim-MW and Sham stim-MW groups: Recorded using 30 Ag/AgCl sintered ring electrodes (Waveguard; ANT-Neuro, Enschede, Netherlands).

Signal processing involved applying a 1–30 Hz bandpass filter to the raw data of all groups. EEG fragments contaminated with artifacts such as electromyograms or eyeblink noise were excluded by visual inspection. After removing the contaminated fragments, residual artifacts were automatically identified and removed using the artifact detector in the LORETA-KEY software (Pascual-Marqui, 2002) https://www.uzh.ch/keyinst/loreta. For the artifact detection and removal pipeline, a subset of features described by Nolan et al. was utilized (Nolan et al., 2010), combined with such as global field power (Lehmann and Skrandies, 1980), to calculate robust z-scores for identifying and removing outliers. Artifact-free 2-s epochs totaling 120 s were adopted for analysis.

### 2.6 iCoh

The analysis of functional localization and effective connectivity of cortical electrical activity was based on cortical signals estimated using standardized low-resolution brain electromagnetic tomography (sLORETA) developed by (Pascual-Marqui, 2002). To evaluate the causal effective connectivity between the regions of interest (ROIs), iCoh implemented in the LORETA-KEY software was used. Following the theoretical framework of (Pascual-Marqui et al., 2014).

The iCoh method is based on a multivariate autoregressive model. Unlike partially directed coherence, which can be influenced by indirect paths and the number of nodes, iCoh isolates the path of interest by setting all irrelevant associations (autoregressive coefficients and noise covariances) other than the direct path of interest to zero. The iCoh value is defined as the squared absolute value of the partial coherence under these constraints and ranges from 0 to 1. This ensures that the measure reflects only direct causal connections, unconfounded by indirect pathways or volume-conduction artifacts. In this study, iCoh values were calculated for the theta (4–8 Hz) and alpha (8–13 Hz) bands.

### 2.7 ROIs

Based on prior literature on the Triple Network Model (Menon, 2011), eleven ROIs corresponding to the DMN, ECN, SN were defined in previous studies related to depression and tDCS (Kaiser et al., 2015; Koizumi et al., 2020; McGrath et al., 2013; Pizzagalli et al., 2018) (Table 1).

• DMN + rACC (five ROIs): Medial Prefrontal Cortex (mPFC), Left Inferior Parietal Lobule (left-IPL), Right Inferior Parietal Lobule (right-IPL), Posterior Cingulate Cortex (PCC), and Rostral Anterior Cingulate Cortex (rACC) as a central hub for integrating emotional information and controlling other brain regions (Allman et al., 2006)
• ECN + rACC (three ROIs): Left Dorsolateral Prefrontal Cortex (left DLPFC), Right Dorsolateral Prefrontal Cortex (right DLPFC), and rACC
• SN + rACC(three ROIs): Left Anterior Insula (left-AI), Right Anterior Insula (right-AI), and rACC

**Table 1:** Regions of interest (ROIs) and their coordinates in the MNI space.

	x	y	z
mPFC	0	55	10
left-IPL	−45	−50	35
right-IPL	45	−50	35
left-DLPFC	−40	26	34
right-DLPFC	40	26	34
left-AI	−30	24	−13.5
right-AI	30	24	−13.5
PCC	0	−50	25
rACC	0	45	0

Abbreviations: IPL, inferior parietal lobule; mPFC, medial prefrontal cortex; PCC, posterior cingulate cortex; rACC, rostral anterior cingulate cortex; DLPFC, dorsolateral prefrontal cortex; AI, anterior insula

### 2.8 Statistical analysis

Statistical analysis focused on the iCoh values within the DMN, ECN, and SN in the theta and alpha bands. iCoh values were log-transformed to approximate a normal distribution. Extreme outliers exceeding three standard deviations (SD), based on the median absolute deviation, were excluded (0.86% of the total data).

Primary Analysis: Multiple regression analysis was conducted with the change in log-transformed iCoh values (post minus pre) as the dependent variable. The independent variables were stimulation (active = 1, sham = 0), intervention (MW = 1, RS = 0), sex (male = 1, female = 0), and age. All independent variables were centered.

Secondary Analysis: For iCoh values showing a significant main effect in the primary analysis, tukey's post-hoc tests were performed with age and sex as covariates to compare differences among the 3 groups (Active stim-RS, Active stim-MW, and Sham stim-MW). The significance level was set at *p < .05*. The Benjamini–Hochberg method was applied to correct *p*-values for multiple testing.

### 2.9 Artificial intelligence assist

The authors utilized Gemini (2026 version) by Google to enhance the readability of the manuscript.

## 3 Results

### 3.1 Demographic characteristics

A total of 77 healthy participants were included in this secondary analysis. The data for the three groups were extracted from the healthy cohorts of the two original studies (CEED 1 and CEED 2). Specifically, data for the Active stim-RS group (*n* = 19) were extracted from the healthy control cohort of the CEED 1 study (Nishida et al., 2019). Data for the Active stim-MW group (*n* = 28) and the Sham stim-MW group (*n* = 30) were derived from the CEED 2 study (Nishida et al., 2021).

The mean age of the participants was 41.69 years, and 55% were female. Table 2 presents a comparison of the sociodemographic variables across the three groups. A significant effect of sex was observed. Regarding age, group comparisons revealed that the Active stim-RS group was significantly younger than the Active stim-MW group (*p = .017)* and the Sham stim-MW group (*p = .023*).

**Table 2:** Demographic data.

	Total (*N* = 77)	Active stim_RS (n = 19)	Active stim_MW (n = 28)	Sham stim_MW (n = 30)	test statistic	*p* value
Sex, n (%)female	42(55%)	7(37%)	16(57%)	19(63%)	χ^2^ = 3.41	0.182
Age, years, M ± SD	41.69 ± 12.49	48.95 ± 15.83	38.29 ± 11.34	40.27 ± 9.28	F = 4.99	0.010
Education period, M ± SD	15.00 ± 1.54	15.63 ± 1.34	14.96 ± 1.63	14.62 ± 1.50	F = 2.61	0.081

Abbreviations: n, sample size; N, total sample size; M, mean; SD, standard deviation; Active stim_RS, Active stimulation-Resting State; Active stim_MW, Active stimulation-Mindfulness Walking; Sham stim_MW, Sham stimulation-Mindfulness Walking

### 3.2 Results of the iCoh analysis and multiple regression

The iCoh values were calculated (Supplementary Table 1), and multiple regression analysis was conducted using the change in log-transformed iCoh values as the dependent variable.

### 3.3 Changes in iCoh within the DMN

**Fig. 1:**
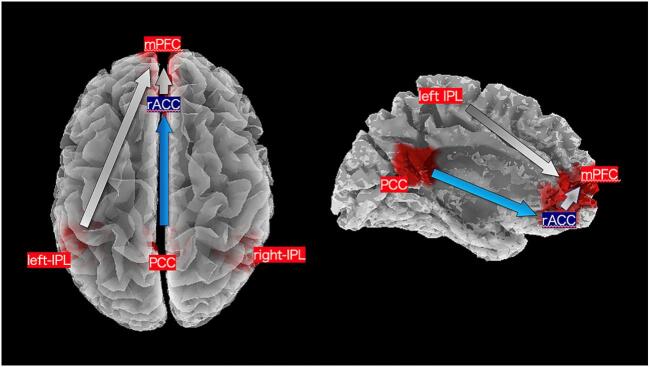
Schematic Summary of iCoh Results in the Default Mode Network: The blue arrow indicates a significant decrease in Active transcranial direct current stimulation (tDCS) compared to sham tDCS, and the gray arrows show a significant decrease in the Mindful walking compared to the resting state (*p* < .05). IPL, inferior parietal lobule; mPFC, medial prefrontal cortex; PCC, posterior cingulate cortex; rACC, rostral anterior cingulate cortex. (For interpretation of the references to colour in this figure legend, the reader is referred to the web version of this article.)

Main Effects of Stimulation and Intervention (Fig. 1) (Supplementary Table 2): Multiple regression analysis revealed a significant main effect of stimulation (active/sham) on the change in iCoh from the PCC to the rACC in DMN-associated hubs. iCoh from PCC to rACC significantly decreased in the Active stimulation condition compared to the Sham stimulation condition (Theta band: *β = −1.603, p = .006;* Alpha band: *β = −1.355, p = .033*). Additionally, a significant main effect of the intervention (MW/RS) was observed for the change in iCoh from rACC to mPFC, where iCoh significantly decreased in the MW condition compared to the RS condition (Theta band: *β = −1.826, p = .033;* Alpha band: *β = −2.121, p = .013).* Furthermore, for the change in iCoh from Left-IPL to mPFC, a main effect of Intervention was observed in the theta band (*β = −1.689, p = .013*), and main effects of both Intervention (*β = −1.844, p = .003*) and Stimulation (*β = −1.027, p = .045*) were observed in the alpha band. No significant differences were observed between other brain interactions.

**Fig. 2:**
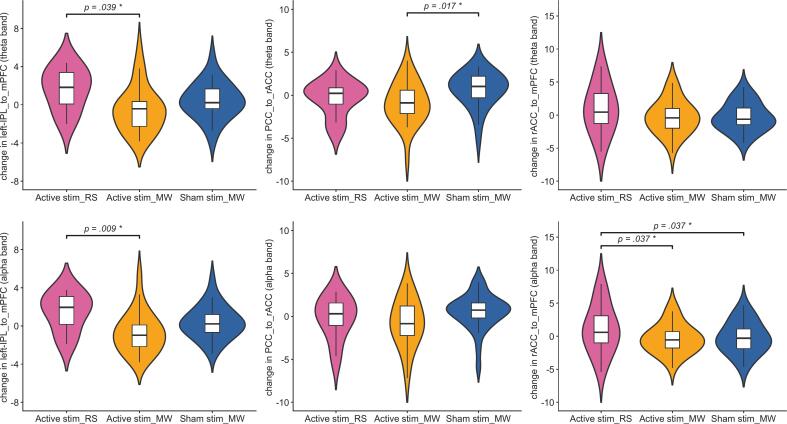
Violin plots of iCoh values in the Default Mode Network: The Active stim-MW group had significantly lower iCoh values for PCC to rACC connectivity in the theta band compared to Sham stim-MW. For rACC to mPFC in the alpha band, the Active stim-MW group showed significantly lower values than Active stim-RS, while Active stim-RS had significantly higher values than Sham stim-MW. The Active stim-MW group also had significantly lower iCoh values from Left-IPL to mPFC in both theta and alpha bands compared to Active stim-RS. *p < .05.* Active stim-RS, Active stimulation-Resting State; Active stim_MW, Active stimulation-Mindful walking; Sham stim_MW, Sham stimulation-Mindful walking; IPL, inferior parietal lobule; mPFC, medial prefrontal cortex; PCC, posterior cingulate cortex; rACC, rostral anterior cingulate cortex.

Between-Group Comparisons (Table 3) (Fig. 2): Secondary analysis results revealed that for the connectivity from the PCC to the rACC in the theta band, the Active stim-MW group exhibited significantly lower iCoh values compared to the Sham stim-MW group (*β = 1.603, p = .017)*.

**Table 3:** Results of Multiple Regression Analysis of Changes in iCoh within the Default Mode Network.

	*β*	SE	*t* value	*p* value
diff left-IPL_to_mPFC (theta band)				
Active_stim RS-Active_stim MW	1.689	0.662	2.552	0.039*
Sham_stim MW-Active_stim MW	0.869	0.550	1.579	0.178
Sham_stim MW-Active_stim RS	−0.820	0.647	−1.268	0.209

diff PCC_to_rACC (theta band)				
Active_stim RS-Active_stim MW	0.283	0.677	0.418	0.677
Sham_stim MW-Active_stim MW	1.603	0.563	2.850	0.017*
Sham_stim MW-Active_stim RS	1.320	0.662	1.995	0.075

diff rACC_to_mPFC (theta band)				
Active_stim RS-Active_stim MW	1.826	0.842	2.169	0.058
Sham_stim MW-Active_stim MW	0.086	0.705	0.122	0.903
Sham_stim MW-Active_stim RS	−1.739	0.826	−2.105	0.058

diff left-IPL_to_mPFC (alpha band)				
Active_stim RS-Active_stim MW	1.844	0.602	3.062	0.009*
Sham_stim MW-Active_stim MW	1.027	0.504	2.037	0.068
Sham_stim MW-Active_stim RS	−0.817	0.590	−1.385	0.171

diff PCC_to_rACC (alpha band)				
Active_stim RS-Active_stim MW	0.327	0.751	0.436	0.664
Sham_stim MW-Active_stim MW	1.355	0.624	2.173	0.099
Sham_stim MW-Active_stim RS	1.028	0.734	1.401	0.248

diff rACC_to_mPFC (alpha band)				
Active_stim RS-Active_stim MW	2.121	0.831	2.554	0.037*
Sham_stim MW-Active_stim MW	0.249	0.695	0.359	0.721
Sham_stim MW-Active_stim RS	−1.872	0.815	−2.296	0.037*

Note: Active stim_RS, Active stimulation-Resting State; Active stim_MW, Active stimulation-Mindfulness Walking; Sham stim_MW, Sham stimulation-Mindfulness Walking; SE, standard error; IPL, inferior parietal lobule; mPFC, medial prefrontal cortex; PCC, posterior cingulate cortex; rACC, rostral anterior cingulate cortex; *, *p* < .05

Regarding the connectivity from the rACC to the mPFC (alpha band), the Active stim-MW group showed significantly lower iCoh values compared to the Active stim-RS group (*β = 2.121, p = .037*). Furthermore, for this alpha band connection, the Active stim-RS group exhibited significantly higher iCoh values than the Sham stim-MW group (*β = −1.872, p = .037*).

Similarly, for the connectivity from the Left-IPL to the mPFC, the Active stim-MW group showed significantly lower iCoh values compared to the Active stim-RS group in both the theta (*β = 1.689, p = .039*) and alpha bands (*β = 1.844, p = .009*).

### 3.4 Changes in iCoh within the ECN

**Fig. 3:**
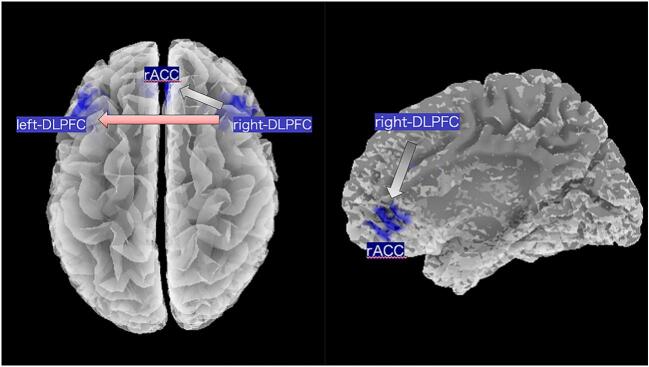
Schematic summary of iCoh results in the Executive Control Network: The red arrow indicates a significantly increased iCoh value in the Active stimulation group compared to the Sham stimulation group. Conversely, the gray arrow shows a significantly decreased iCoh value in the Mindful walking compared to the Resting State (p < .05). rACC, rostral anterior cingulate cortex; DLPFC, dorsolateral prefrontal cortex. (For interpretation of the references to colour in this figure legend, the reader is referred to the web version of this article.)

Main Effects of Stimulation and Intervention (Fig. 3) (Supplementary table 3). A significant main effect of stimulation was observed for the change in iCoh from the right to the Left-DLPFC in correlated ECN hubs. iCoh flow from the right to left DLPFC significantly increased in the Active stimulation condition compared to the Sham stimulation condition (theta band: *β = 1.479, p = .012;* alpha band: *β = 1.197, p = .027)*. On the other hand, for the change in iCoh from Right-DLPFC to rACC (alpha band), a significant main effect of Intervention was observed, with iCoh significantly decreasing in the MW condition compared to the RS condition (*β = −1.440, p = .046*). No significant differences were observed in the other interactions.

**Fig. 4:**
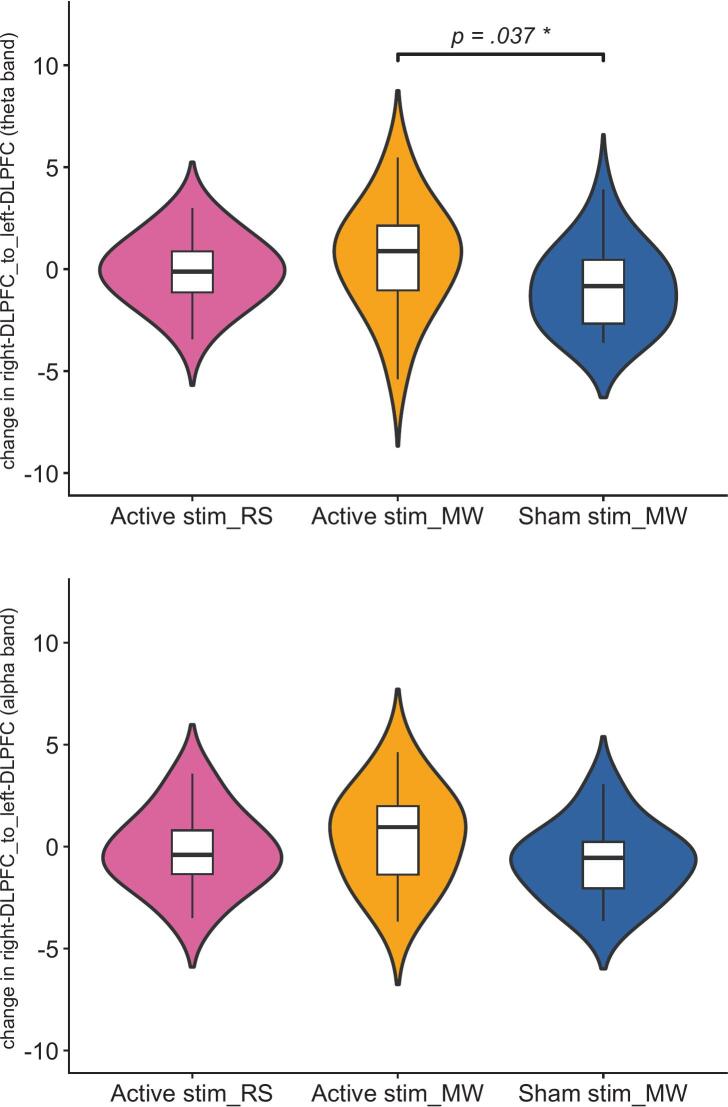
Violin Plot of the Executive Control Network: The Active stim-MW group had significantly higher iCoh values from Right-DLPFC to Left-DLPFC in the theta band compared to the Sham stim-MW group. Active stim-RS, Active stimulation-Resting State; Active stim_MW, Active stimulation-Mindful walking; Sham stim_MW, Sham stimulation-Mindful walking; DLPFC, dorsolateral prefrontal cortex; rACC, rostral anterior cingulate cortex.

Between-Group Comparisons (Table 4) (Fig. 4): For iCoh from Right-DLPFC to Left-DLPFC (theta band), the Active stim-MW group showed significantly higher values compared to the Sham stim-MW group (*β = −1.479, p = .037)*.

**Table 4:** Results of Multiple Regression Analysis of Changes in iCoh within the Executive Control Network.

	*β*	SE	*t* value	*p* value
diff right-DLPFC_to_rACC (theta band)				
Active_stim RS-Active_stim MW	1.401	0.722	1.941	0.169
Sham_stim MW-Active_stim MW	0.473	0.604	0.783	0.436
Sham_stim MW-Active_stim RS	−0.928	0.706	−1.314	0.290

diff right-DLPFC_to_left-DLPFC (theta band)				
Active_stim RS-Active_stim MW	−0.631	0.688	−0.918	0.362
Sham_stim MW-Active_stim MW	−1.479	0.575	−2.570	0.037*
Sham_stim MW-Active_stim RS	−0.848	0.676	−1.254	0.321

diff right-DLPFC_to_rACC (alpha band)				
Active_stim RS-Active_stim MW	1.440	0.708	2.035	0.137
Sham_stim MW-Active_stim MW	0.662	0.593	1.117	0.268
Sham_stim MW-Active_stim RS	−0.778	0.693	−1.123	0.268

diff right-DLPFC_to_left-DLPFC (alpha band)				
Active_stim RS-Active_stim MW	−0.611	0.639	−0.956	0.352
Sham_stim MW-Active_stim MW	−1.197	0.531	−2.253	0.082
Sham_stim MW-Active_stim RS	−0.586	0.625	−0.937	0.352

Note: Active stim_RS, Active stimulation-Resting State; Active stim_MW, Active stimulation-Mindfulness Walking; Sham stim_MW, Sham stimulation-Mindfulness Walking; SE, standard error; rACC, rostral anterior cingulate cortex; DLPFC, dorsolateral prefrontal cortex; *, p < .05.

### 3.5 Changes in iCoh within the SN

Regarding the iCoh within the SN, no significant main effects of Stimulation or Intervention were observed (Supplementary Table 4).

## 4 Discussion

To our knowledge, this study is the first to investigate the effects of concurrent tDCS and mindful walking on the causal iCoh within brain networks. tDCS and mindful walking reconfigure brain networks via complementary mechanisms: tDCS enhances ECN connectivity and suppresses posterior-to-anterior DMN coupling, while mindfulness reduces especially anterior DMN and ECN connectivity.

Recent findings suggest that the DMN is not unitary but divides into two functional subdivisions: an anterior network supporting emotion regulation and decision making, and a posterior network supporting sensory integration (Vidaurre et al., 2018). In the present study, active tDCS significantly decreased the iCoh from the PCC to the rACC in both the theta and alpha bands. Interpreting this result from the viewpoints of (Vidaurre et al., 2018) and the mind-wandering dynamic framework proposed by (Christoff et al., 2016), the PCC acts as a posterior hub that integrates memories and contextual information. Consequently, attenuation of information flow from the PCC to the rACC, where it is involved in emotional processing, can be interpreted as a mechanism that inhibits the influx of maladaptive memories and negative contexts into the current emotional state. (Hamilton et al., 2011) and (Kaiser et al., 2015) linked excessive DMN connectivity in patients with major depression with persistent negative self-reflection and rumination. Notably, rumination is recognized as a transdiagnostic vulnerability factor that maintains both depression and anxiety. This finding suggests that tDCS neurophysiologically facilitates cognitive decoupling from experience, which is the primary goal of mindfulness.

However, the MW alone significantly decreased the directional information flow from the rACC to the mPFC. Neurobiologically, the mPFC has been established as a core hub of the DMN involved in self-referential processing (Christoff et al., 2016), whereas the rACC is generally involved in processing emotions and plays a central role in controlling other brain networks through functional synchronization with the salience network (Minami et al., 2022). Therefore, the reduced effective connectivity from the rACC to the mPFC suggests that the MW may suppress the mechanism by which the SN triggers DMN activity. Consistent with the notion that mindfulness improves attentional regulation (Malinowski, 2013), this result implies that focused attention on walking shields the mPFC from emotional or conflict signals arising from the rACC, effectively preventing the initiation of ruminative processing.

The present results demonstrate that active tDCS significantly increases the directional iCoh from the right to the left DLPFC in theta band. Anodal stimulation has been widely considered to enhance cortical excitability (Nitsche and Paulus, 2000). However, recent evidence highlights that the neuromodulatory effects of tDCS are highly complex and influenced by various other factors beyond stimulation polarity. These factors include individual anatomical variations that widely disperse the electric field beyond the targeted DLPFC (Yachou et al., 2025), diverse stimulation parameters modulating prefrontal cognitive networks (Meinzer et al., 2024), and alterations in the balance between excitatory and inhibitory neurotransmission within these regions (Yamada and Sumiyoshi, 2021). Considering these nuanced mechanisms, rather than a simple local excitation, the observed increase in information flow from the contralateral right DLPFC to the stimulated left DLPFC in the present study suggests that tDCS facilitated large-scale interhemispheric communication, thereby promoting the functional integrity of the ECN. Strengthening the ECN is directly linked to the capacity to exert top-down control, enabling disengagement from mind-wandering, and sustaining attention on the present moment (Hasenkamp et al., 2012; Malinowski, 2013).

Furthermore, the Active stim-MW group exhibited significantly lower directional connectivity from the right DLPFC to the rACC compared to the Sham stim-MW group.

The observed reduction in information flow from the right DLPFC to the rACC generally aligns with the ‘neural efficiency’ hypothesis originally proposed by (Haier et al., 1988) and applied to the combination of mindfulness and tDCS by (Hunter et al., 2018). By externally boosting DLPFC excitability, active tDCS may diminish the necessity of effortful top-down control signals to the SN. This interpretation is supported by previous research indicating that efficient attentional processing is associated with reduced input from the right prefrontal cortex to the anterior cingulate cortex (Kitaura et al., 2017) and that the DLPFC plays a critical role in the top-down regulation of emotion and attention (Kaiser et al., 2015). Thus, our findings suggest that tDCS may have externally supplemented the attentional resources required for mindfulness, potentially allowing participants to maintain a mindful state at a lower neural cost.

However, this study had several limitations. First, although significant group differences in age and sex were observed, these variables were included as covariates in the regression analysis to control for potential confounding effects. Second, this study focused on the immediate effects of a single session; thus, the cumulative effects and long-term neuroplasticity resulting from repeated sessions were not evaluated. Third, because this study is a post-hoc secondary analysis extracting and reorganizing data from two distinct trials, it was not originally designed as a single, fully randomized parallel-group trial. Although we structured the data to enable a three-group comparison, we cannot completely rule out the influence of unmeasured cohort-specific confounding factors.

Fourth, as a practical consequence of combining these two datasets, there were methodological differences in EEG data acquisition. Although both studies utilized the same EEG amplifier, EEG caps from the same manufacturer, and the same recording room, the CEED 1 study recorded from 64 channels, whereas the CEED 2 study recorded from 30 channels due to the practical constraints of treadmill walking. We acknowledge that this discrepancy in the number of recorded channels could act as an additional confounding factor when comparing the groups.

These limitations highlight the need for further research. Importantly, findings from this fundamental study suggest that the effects of non-invasive brain stimulation can vary significantly depending on the specific stimulation conditions and concurrent behavioral states. Therefore, future studies must carefully control these parameters while conducting longitudinal trials involving clinical populations to evaluate the actual therapeutic efficacy and cumulative effects.

In conclusion, the concurrent application of tDCS and mindful walking synergistically facilitates brain network reconfiguration by suppressing DMN hyperactivity and enhancing ECN connectivity. This dual approach offers a promising clinical strategy to correct pathological network imbalances, highlighting the importance of optimizing stimulation conditions to maximize therapeutic outcomes in psychiatric disorders.

## Declaration of competing interest

The authors declare that they have no known competing financial interests or personal relationships that could have appeared to influence the work reported in this paper.

## Appendix A Supplementary Data

Supplementary material 1 file.

Supplementary material 2 Results of the Multiple Regression Analysis of Changes in iCoh withinthe Default Mode Network.

Supplementary material 3 Results of the Multiple Regression Analysis of Changes in iCoh within the Excective Control Network.

Supplementary material 4 Results of the Multiple Regression Analysis of Changes in iCoh within the Salience Network.
